# Myeloid maturation potentiates STAT3-mediated atypical IFN-γ signaling and upregulation of PD-1 ligands in AML and MDS

**DOI:** 10.1038/s41598-019-48256-4

**Published:** 2019-08-12

**Authors:** Digdem Yoyen-Ermis, Gurcan Tunali, Ece Tavukcuoglu, Utku Horzum, Didem Ozkazanc, Tolga Sutlu, Yahya Buyukasik, Gunes Esendagli

**Affiliations:** 1 Department of Basic Oncology, Hacettepe University Cancer Institute, Ankara, Turkey; 2 Nanotechnology Research and Application Center, Sabanci University, Istanbul, Turkey; 3 Department of Hematology, Faculty of Medicine, Hacettepe University, Ankara, Turkey; 4 Present Address: Lokman Hekim University, Faculty of Medicine, Department of Medical Biology, Ankara, Turkey

**Keywords:** Tumour immunology, Immunoediting

## Abstract

Interferon (IFN)-γ is the major mediator of anti-tumor immune responses; nevertheless, cancer cells use intrigue strategies to alter IFN-γ signaling and avoid elimination. Understanding the immune regulatory mechanisms employed by acute myeloid leukemia (AML) and myelodysplastic syndrome (MDS) cells upon exposure to IFN-γ is critical for development of immunotherapy and checkpoint blockade therapy approaches. This study aims to explore the influence of myeloid maturation on IFN-γ-induced PD-L1 and PD-L2 expression and on pro-leukemogenic transcription factor STAT3 signaling in AML and MDS. Stimulation of myeloid blasts’ maturation by all-trans retinoic acid (ATRA) or 1α,25-dihydroxyvitamin D3 (vitamin D) increased the CD11b^+^ fraction that expressed PD-1 ligands in response to IFN-γ. Intriguingly, STAT3 pathway was potently induced by IFN-γ and strengthened upon prolonged exposure. Nonetheless, STAT3-mediated atypical IFN-γ signaling appeared as a negligible factor for PD-L1 and PD-L2 expression. These negative influences of IFN-γ could be alleviated by a small-molecule inhibitor of STAT3, stattic, which also inhibited the upregulation of PD-L1. In conclusion, induction of myeloid maturation enhances the responsiveness of AML and MDS cells to IFN-γ. However, these malignant myeloid cells can exploit both STAT3 pathway and PD-1 ligands to survive IFN-γ-mediated immunity and maintain secondary immune resistance.

## Introduction

Regulatory failures in T-cell mediated anti-tumor immune responses, which correlate with disease progression and severity, are widely observed in acute myeloid leukemia (AML) and myelodysplastic syndrome (MDS)^1,2^. Cells of myeloid origin are amongst the key partners of T-cells; resulting in the increased likelihood of transformed myeloid cells to directly encounter with the tumor-reactive T-cells^3^. Nevertheless, in response to anti-tumor reactions, malignant cells use precise mechanisms, renowned as adaptive or secondary immune resistance, to evade immune elimination^4,5^. Intriguingly, AML and MDS blasts can express costimulatory molecules such as B7-1 (CD80), B7-2 (CD86), and B7-H2 (ICOS-LG) which normally take part in cancer immunity; nonetheless, these malignant cells survive immune reactions through acquisition of adaptive resistance which is essentially mediated by IFN-γ^5–7^. Even though the anti-tumor activities of IFN-γ are well-acknowledged, this cytokine can also initiate regulatory feedback mechanisms to avoid collateral damage and to maintain homeostasis^8,9^. Inhibitory molecules such as programmed death ligand-1 (PD-L1) and PD-L2 are upregulated in response to IFN-γ^8,10–12^. It is not unusual to identify tumors that hijack this homeostatic mechanism to evade immune reactions^13^. Even though the signaling pathways induced by IFN-γ are rather complex, the expression PD-1 ligands is mainly controlled by the Janus kinase 1 (JAK1) and JAK2, the signal transducers and activators of transcription 1 (STAT1), and the interferon-regulatory factor 1 (IRF1) pathways^8,11,12,14^. In various cell types, including cancer cells and myeloid cells, STAT3 can be induced as an atypical signal transducer of IFN-γ while the interplay between STAT1 and STAT3 determines the biological outcome^14–18^. Nevertheless, the role of STAT3 in the regulation of PD-1 ligands has been a controversy. In AML and MDS, STAT3 serves as a pro-leukemogenic transcription factor which is associated with bad prognosis and short disease-free survival^19–22^. Accordingly, STAT3 inhibition has been regarded as a promising therapeutic approach for leukemia^20,23^.

Currently, the treatment of myeloproliferative diseases includes conventional chemotherapy and/or induction of myeloid maturation by ATRA which is successfully applied in acute promyleocytic leukemia (APL)^24^. In addition, potential of immunotherapy, including the PD-1 checkpoint blockade in myeloproliferative diseases is yet to be fully developed^25^. Anti-tumor immune reactions are recovered by the blockade of PD-1 pathway and subsequent therapeutic benefits are achieved in certain non-hematopoietic solid tumors and in Hodgkin’s lymphoma^26^.

The present study aims to investigate PD-1 ligands’ upregulation and STAT3 activation as secondary immune resistance mechanisms elicited by AML and MDS cells upon exposure to IFN-γ under the maturation pressure by ATRA or D3 treatment. Here, we demonstrate that the ATRA- or D3-induced maturation of primary AML or MDS cells and cell lines promotes the IFN-γ-induced upregulation of PD-1 ligands and activation of STAT3 pathway. STAT3 had a minor influence directly on PD-L1 expression. Treatment with stattic, a small-molecule alleged for STAT3 inhibition, interfered both with STAT3 and STAT1 activation, and hampered IFN-γ-induced PD-L1 expression in AML or MDS.

## Results

### ATRA- or D3-induced myeloid maturation in AML and MDS yields a CD11b^+^ sub-population with a high capacity to upregulate PD-1 ligands upon IFN-γ treatment

**Figure 1:**
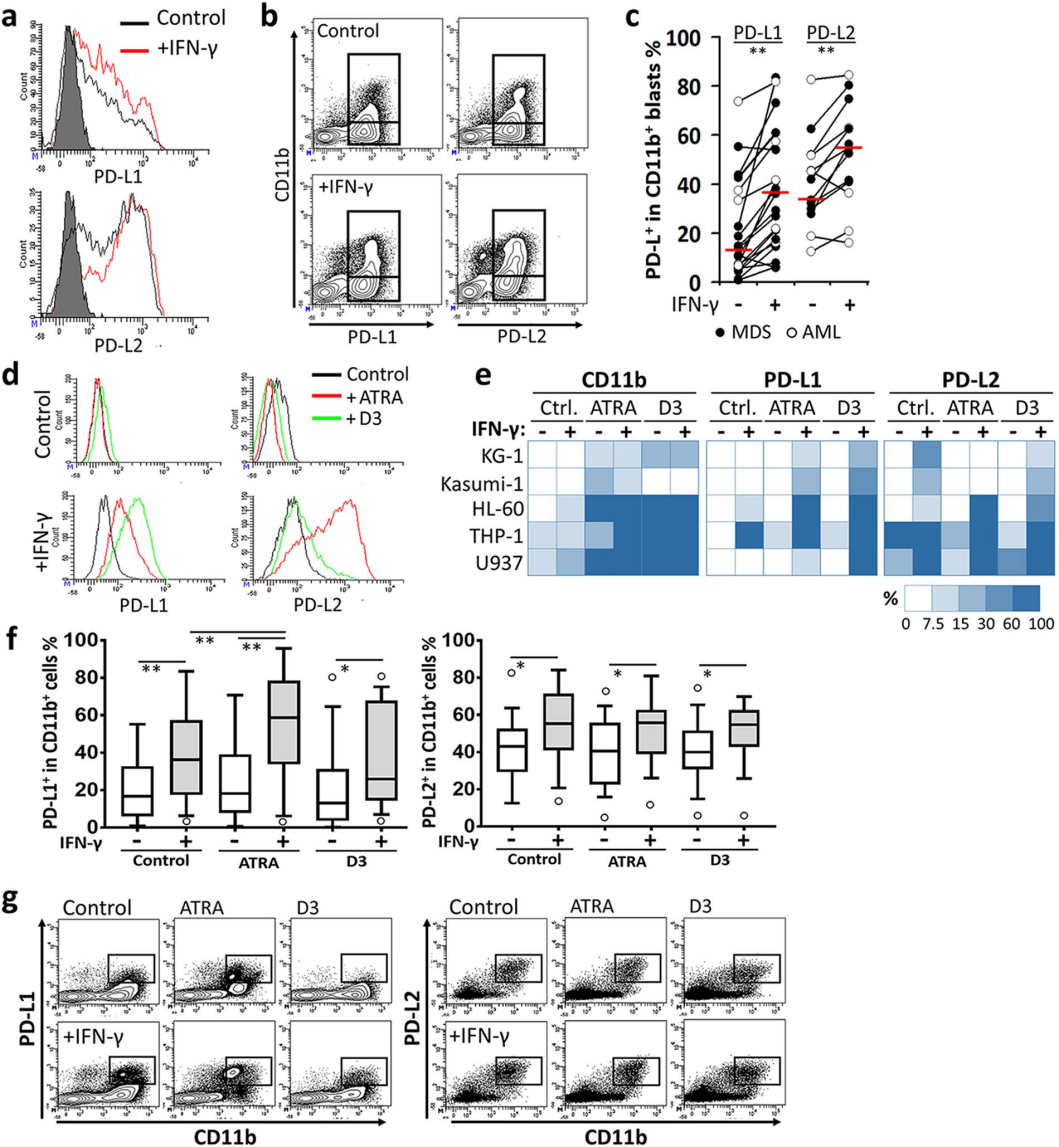
CD11b^+^ MDS and AML cells have increased capacity to express PD-1 ligands in response to IFN-γ. Patient-derived myeloid blasts or myeloid leukemia cell lines were stimulated with IFN-γ for 48 h. The expression of PD-L1 and PD-L2 was assessed by flow cytometry (**a**) without or (**b**) with CD11b gating. Representative plots are given. (**c**) Distribution of MDS and AML patients according to the change in the percentage of CD11b^+^ blasts with PD-1 ligand expression following the treatment with IFN-γ. (**d**) Representative flow cytometry histograms of PD-1 ligands are shown for HL-60 cell line pretreated with ATRA or D3. (**e**) Expression levels of CD11b, PD-L1, and PD-L2 following the treatment with ATRA or D3 and/or IFN-γ were schematically shown as a heat-map from the panel of myeloid leukemia cell lines screened (also refer to Supplementary Fig. 3A,D) (**f**) The myeloid blasts from AML or MDS patients’ bone marrow aspirates were pretreated with ATRA or D3; then, the percentage of IFN-γ-induced PD-L1 or PD-L2 expression was determined. Outliers are shown as empty circles. (**g**) Flow cytometry counter-plots for PD-1 ligands and CD11b are shown from a representative patient sample which was pretreated with ATRA or D3 prior to IFN-γ stimulation. (*P < 0.05, **P < 0.01; for cell lines, *n* ≥ 3; patient samples, *n* = 30).

CD11b levels are related to myeloid maturation, however, it has been associated with bad prognosis in the myeloproliferative diseases^27–30^. Consistent with previous reports^6,7^, when stimulated with IFN-γ, patient-derived AML or MDS cells expressed higher levels of PD-L1 and PD-L2 (Fig. 1a). Additionally, CD11b^+^ blasts were identified with an explicit capacity to upregulate PD-1 ligands in 26/30 patients (Fig. 1b,c). This CD11b^+^ subpopulation was the major fraction that increased the overall frequency of PD-L1- or PD-L2-positive cells (77.09 ± 18% in total PD-L1^+^ blasts, 55.11 ± 19.38% in total PD-L2^+^blasts) in patient bone marrow aspirates. In terms of PD-1 ligands’ upregulation, there was no statistically significant difference between AML and MDS patients (Fig. 1c).

Next, we asked if preferential expression of PD-1 ligands on CD11b^+^ blasts was associated with myeloid maturation. For this purpose, the derivatives of vitamin A and vitamin D (ATRA and D3, respectively) were used as well-known inducers of myeloid differentiation in leukemia cells^31,32^. First, the usefulness of CD11b to distinguish the myeloid cells with advanced maturation was tested on HL-60 cell line. In comparison to other myeloid markers (CD11c, CD14, CD15, CD16, CD66b, and HLA-DR), CD11b displayed consistent expression kinetics and reached to the highest level at 96 h (control, 3 ± 1.9%; with ATRA, 62.03 ± 4.61%; with D3, 74.48 ± 6.41%) (Supplementary Fig. 1A,B). CD11b^hi^ cells were also frequent in the high density-gradient fraction as a facet of maturation and differentiation (Supplementary Fig. 2A,B). A panel of myeloid leukemia cell lines found at different maturation stages according to French-American-British (FAB) classification were used to further support the data. All myeloid leukemia cell lines studied responded to ATRA or D3 and increased the expression of CD11b (expression change range, 6.2–20.7 fold) (Fig. 1d and Supplementary Fig. 3A). The cells with particular immature characters, KG-1 and Kasumi-1^33^, presented a small CD11b^+^ sub-population. IFN-γ stimulation did not negatively affect the expression of CD11b (Fig. 1e and Supplementary Fig. 3A).

Expectedly, the panel of leukemia cell lines used had a certain heterogeneity in their basal expression of PD-1 ligands and responsiveness to IFN-γ. Myeloid maturation with ATRA or D3 did not affect the basal expression of PD-1 ligands. On the other hand, PD-L1 and PD-L2 were upregulated especially on the CD11b^+^ cells when exposed to IFN-γ (Fig. 1e and Supplementary Fig. 3B–F). Correspondingly, the IFN-γ-induced CD11b^+^ cells displayed a reduced capacity to co-stimulate T-cells whereas the blockade of PD-1 ligands significantly augmented T-cell proliferation (see Supplementary Fig. 4A,B).

In the majority of the patients (60%), irrespective of AML or MDS diagnosis, treatment with ATRA or D3 significantly enhanced CD11b expression (increment range, 25–170%). In response to IFN-γ, CD11b^+^ AML or MDS blasts significantly upregulated PD-L1 and PD-L2 (Fig. 1f,g, and Supplementary Fig. 5). PD-L2 and especially PD-L1 expression were correlated with CD11b (PD-L1, r = 0.406, *P* = 0.025; PD-L2, r = 0.42, *P* = 0.02, *n* = 30). The majority of PD-L1- or PD-L2-expressing cells were determined as of the CD11b-high sub-population. Accordingly, not only under the influence of IFN-γ but also under basal conditions, the blasts with high CD11b expression tend to carry higher levels of PD-1 ligands (Supplementary Fig. 6).

Collectively, CD11b was identified as a surrogate marker that indicates the increased tendency of AML and MDS cells to display PD-L1 and PD-L2. Myeloid maturation induced by ATRA or D3 enhanced the capacity of CD11b^+^ blasts to upregulate PD-1 ligands in response to IFN-γ.

### STAT3-mediated atypical IFN-γ signal transduction is sustained in myeloid leukemia cells

**Figure 2:**
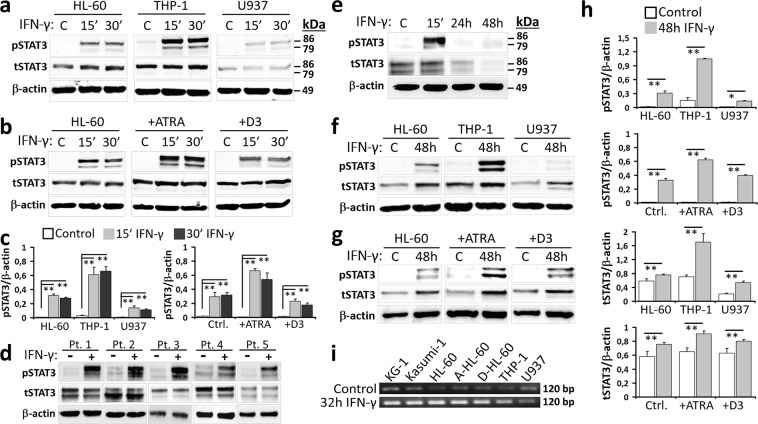
STAT3 pathway is induced and strengthened by IFN-γ in malignant myeloid cells. Upon stimulation with IFN-γ, total STAT3 and Tyr705 phosphorylated pSTAT3 levels were evaluated by Western-Blot in (**a**,**c**) myeloid leukemia cell lines, (**b**,**c**) HL-60 cells pretreated with ATRA or D3, (**d**) patient-derived cells from the bone marrow aspirates, and (**e**) myeloid cells from the peripheral blood of healthy donors. Total STAT3 and pSTAT3 levels in (**f**,**h**) control myeloid leukemia cell lines or (**g**,**h**) ATRA- or D3-tretaed HL-60 cells under prolonged (48 h) exposure to IFN-γ. (**a**,**b**,**d–g**) Representative Western-Blot images are given. Please note that the blots from each cell line are shown as cropped from different parts of the same gel. (**c**,**h**) In order to obtain semi-quantitative data, band intensities of total or phosphorylated STAT3 were normalized with those of the house-keeping β-actin protein. Semi-quantitative data of total STAT3 levels upon stimulation with IFN-γ for 15 or 30 minutes are shown in Supplementary Fig. 7. (**i**) STAT3 gene expression was studied by RT-PCR in control or IFN-γ-treated (32 h) myeloid leukemia cell lines. Representative agarose gel electrophoresis images are shown. (*P < 0.05, **P < 0.01; for cell lines, *n* ≥ 3; patient or healthy control samples, *n* = 3; A-HL-60 and D-HL-60, HL-60 cells treated with ATRA and D3, respectively).

STAT3 is a critical transcription factor that favors leukemogenesis^19,23^. Depending on the cell type, IFN-γ may induce STAT3 as an alternative pathway^15^ and modulate the expression of PD-1 ligands^11^. In myeloid leukemia cells under steady state conditions, STAT3(Tyr705) phosphorylation (pSTAT3) was negligible, indicating no constitutive activation of this pathway. Transient exposure to IFN-γ resulted in a significant upsurge of pSTAT3 (Fig. 2a,c). The promotion of myeloid maturation with ATRA, but not with D3, strengthened STAT3 activation (Fig. 2b,c). In addition, when the patient-derived CD11b^+^ AML and MDS samples were treated with IFN-γ, pSTAT3 was significantly induced (Fig. 2d). STAT3 pathway in CD11b^+^ myeloid cells from healthy donors’ peripheral blood also responded to IFN-γ (Fig. 2e). As a general rule, cytokine-induced signaling cascades are short-lived in order to regulate the transient cellular responses^34^. Intriguingly, the extended exposure to IFN-γ prolonged the activation of STAT3 and upregulated its mRNA and total protein expression in myeloid leukemia cells (Fig. 2f–i). In order to test if IFN-γ induces the production of paracrine or autocrine factors leading to an indirect increase in pSTAT3, secretory protein transport was inhibited in HL-60 and THP-1 cells, and then these cells were stimulated with IFN-γ. The results confirmed a direct stimulation of STAT3 pathway by IFN-γ (Supplementary Fig. 8). In addition, either in control or IFN-γ-stimulated cells (i.e. THP-1) no common cytokines such as IL-6, IL-10, and GM-CSF which induce STAT3 pathway was detected (data not shown).

In contrast to malignant myeloid cells, STAT3 pathway was only transiently reinforced and both the total STAT3 and pSTAT3 levels were diminished in healthy donors’ myeloid cells following IFN-γ exposure for 24 h and 48 h (Fig. 2e). On the other hand, STAT3 gene expression was upregulated in these healthy CD11b^+^ cells (Supplementary Fig. 9).

### Stattic, a small-molecule inhibitor of STAT3, interferes with IFN-γ-induced PD-L1 expression in AML and MDS

In order to better dissect the role of IFN-γ-induced STAT3 activation in the upregulation of PD-1 ligands on myeloid leukemia cells, a small-molecule inhibitor of STAT3 pathway, namely stattic, was used^35^. Stattic preferentially blocks the phosphorylation and dimerization of STAT3 but also possesses the potential to interfere with STAT1 in a context- and time-dependent manner^35,36^. Accordingly, THP-1, ATRA- or D3-treated HL-60, and U937 cells which were identified with the highest PD-1 ligand expression capacity, were treated with stattic prior to incubation with IFN-γ.

**Figure 3:**
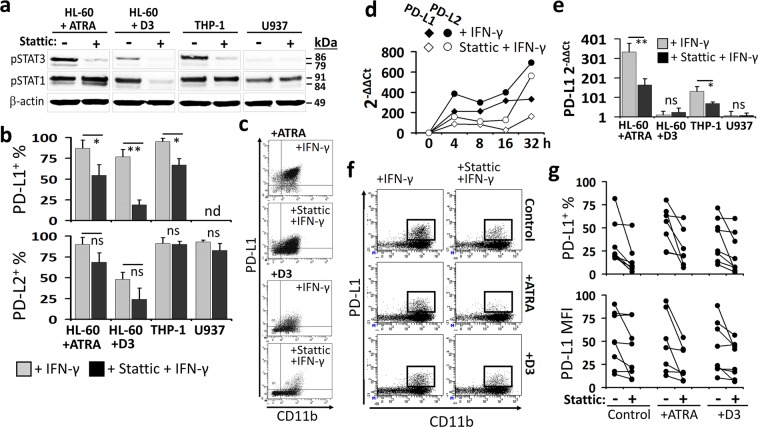
Interfering the STAT1/STAT3 pathway with the small-molecule stattic hinders the IFN-γ-induced upregulation of PD-1 ligands in myeloid leukemia cells. ATRA- or D3-treated HL-60, THP-1, and U937 which displayed high capacity to express PD-1 ligands were used as representative myeloid leukemia cell lines. (**a**) Prior to induction with IFN-γ (for 15 min.), these cells were exposed to stattic and the change in pSTAT3 (Tyr705) and pSTAT1 (Tyr701) levels were assayed by Western-Blot. Please note that the blots from each cell line or patient sample are shown as cropped from different parts of the same gel. (**b**) The effect of stattic on the percentage PD-L1^+^ or PD-L2^+^ myeloid leukemia cells following 24 h stimulation with IFN-γ. (**c**) Representative flow cytometry dot plots are shown for the ATRA- or D3-treated HL-60 cells. The change in PD-L1 and PD-L2 mRNA levels in the IFN-γ-induced (**d**) ATRA-treated HL-60 cells (at 4, 8, 16, and 32 h) and (**e**) other myeloid leukemia cell lines (for PD-L1 at 32 h) with or without the stattic pretreatment was studied by real-time RT-PCR. (**f**,**g**) The effect of stattic on IFN-γ-induced PD-L1 expression (at 24 h) on the CD11b^+^ blasts from the control, ATRA- or D3-treated AML or MDS patent samples; (**f**) representative flow cytometry plots and (**g**) the percentage of PD-L1^+^ cells with or without stattic treatment are given. (*P < 0.05, **P < 0.01; for cell lines, n ≥ 3; patient samples, n = 7; ns, not significant).

**Figure 4:**
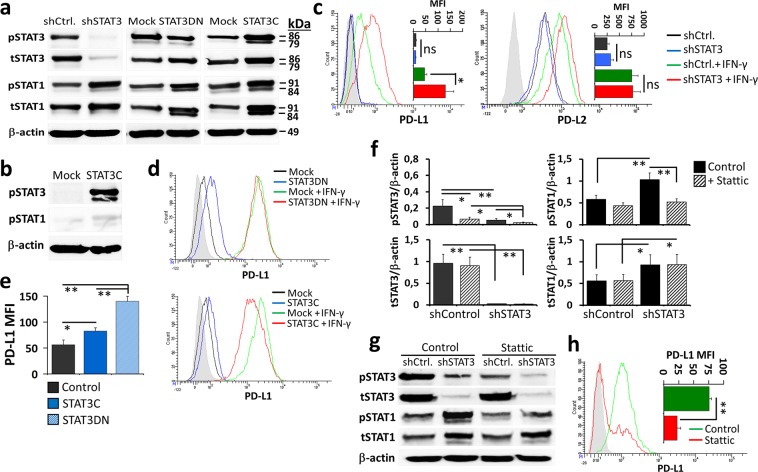
Dual effects of STAT3 on the IFN-γ-induced PD-L1 expression in myeloid leukemia cells. STAT3 expression was modulated in THP-1 as a representative CD11b^+^ myeloid leukemia cell line with a high capacity to upregulate PD-L1 in response to IFN-γ. In THP-1 cells, STAT3 was knocked-down with a short hairpin RNA (shRNA) construct (shSTAT3), or its transcriptional activities were hindered with an expression cassette encoding for the double-negative STAT3 (STAT3DN) molecule. The STAT3 protein which undergoes constitutive phosphorylation (STAT3C) was also introduced. Transductions with a control shRNA construct or empty vectors (mock) were also performed as controls. (**a**) The outcomes of genetic modification of STAT3 pathway were assessed by Western-Blot analyses in THP-1 cells stimulated with IFN-γ (15 min.). Typical results for total and phosphorylated STAT3 and STAT1 are given. Please note that the blots from each cell line are shown as cropped from different parts of the same gel. (**b**) pSTAT3 (Tyr705) and pSTAT1 (Tyr701) levels in the mock- or STAT3C-transduced THP-1 cells without IFN-γ induction. (**c**) Flow cytometry overlay histograms and median fluorescence intensity (MFI) graphs for basal or IFN-γ-induced PD-L1 and PD-L2 expression (24 h) on the THP-1 cells genetically-modified with shControl or shSTAT3. In the THP-1 cells genetically-modified with STAT3DN or STAT3C, (**d**) flow cytometry overlay histograms for the IFN-γ-induced PD-L1 expression (at 24 h) and (**e**) MFI graphs for the steady state PD-L1 levels were determined. (**f**) In the STAT3 knocked-down (shSTAT3-transduced) cells, the effect of stattic on the IFN-γ-induced phosphorylation of STAT3 and STAT1 (15 min.) is studied by Western Blot. Representative protein bands are given in (**g**). (**h**) shSTAT3/THP-1 cells were pretreated with stattic and stimulated with the IFN-γ (24 h) and PD-L1 expression was determined by flow cytometry. (*P < 0.05, **P < 0.01; n ≥ 3; shCtrl., shControl; ns, not significant).

Stattic significantly reduced the levels pSTAT3 whereas pSTAT1 was also differentially affected (Fig. 3a and Supplementary Fig. 10). Under the influence of stattic pre-treatment, IFN-γ-induced STAT1 pathway was strengthened in ATRA-treated HL-60 cells. However, in THP-1 and D3-treated HL-60 cells pSTAT1 levels had a tendency to decrease. On the other hand, STAT1 phosphorylation was not influenced in U937 which was the least responsive cell line to IFN-γ (Fig. 3a and Supplementary Fig. 10). When the stattic-pretreated myeloid cells were stimulated with IFN-γ for 24 h, which was a sufficient period to upregulate PD-1 ligands (Supplementary Fig. 11), the percentage of PD-L1^+^ cells was considerably decreased (Fig. 3b,c). PD-L2 expression was declined in the ATRA- or D3-treated HL-60 cells albeit not reaching to the level of statistical significance (Fig. 3b). The IFN-γ-induced upregulation of PD-L1 and PD-L2 gene expression was also hindered when the cells were under the influence of stattic. Nevertheless, PD-L2 mRNA level was more rapidly restored than that of PD-L1 (Fig. 3d). At 32 h, PD-L1 gene expression was still suppressed by stattic in ATRA-treated HL-60 and THP-1 cells whereas it was restored in the D3-treated HL-60 cells and in U937 cells (Fig. 3e). Moreover, the pretreatment with stattic reduced the IFN-γ-induced upregulation of PD-L1 on the patient-derived CD11b^+^ AML or MDS blasts (Fig. 4f,g, and Supplementary Fig. 12).

Overall, in myeloid leukemia cells, stattic not only abrogated the IFN-γ-induced STAT3 activation but also interfered with STAT1 phosphorylation. Thus, this small-molecule was determined as a STAT3-biased modulator of the IFN-γ-induced STAT1/STAT3 pathway and hampered the upregulation of PD-1 ligands, especially PD-L1.

### STAT3 has a minor impact on PD-L1 expression in myeloid leukemia cells

In order to better recognize the influence of STAT3 pathway on PD-1 ligand expression in myeloid leukemia cells, STAT3 activity was modulated in THP-1, which was selected as a representative cell line according to the results obtained in the previous assays. STAT3 expression was knocked-down or its activity was impeded by introducing shSTAT3 or STAT3DN. THP-1 cells were also genetically-modified to express a constitutively active form of STAT3, STAT3C. In response to IFN-γ, pSTAT3 levels were reduced in the STAT3DN-modified but enhanced in the STAT3C-modified cells (Fig. 4a). Expectedly, this pathway was almost totally abrogated in the cells transduced with shSTAT3. Nevertheless, in all three cell types with modified STAT3, both total STAT1 expression and pSTAT1 levels were increased (Fig. 4a). STAT3 activation did not directly influence STAT1 activation since pSTAT1levels were not changed without IFN-γ stimulation  in STAT3C-modified cells (Fig. 4b). These data may indicate an indirect influence of STAT3 on STAT1 activity which was observed only in the presence of IFN-γ. A compensatory interplay between STAT1 and STAT3 was previously reported in several other cell types^37^.

Knocking STAT3 levels down significantly increased the IFN-γ-induced expression of PD-L1 whereas PD-L2 was not affected (Fig. 4c). On STAT3DN/THP-1 cells and, to a smaller extent, on STAT3C/THP-1 cells, PD-L1 levels were enhanced even in the absence of IFN-γ stimulation (Fig. 4d,e). Intriguingly, under the influence of IFN-γ, this modulatory effect by STAT3DN or STAT3C on PD-L1 expression was abrogated. Alternatively, we stimulated THP-1 cells with IL-6, a cytokine which directly stimulates the STAT3 pathway, alone or in combination with IFN-γ. IL-6 alone could not upregulate PD-L1 and the increment in PD-L1 expression did not reached to the level of statistical significance when IL-6 and IFN-γ were combined (Supplementary Fig. 13). Next, in order to better inspect the downregulation of PD-L1 obtained with stattic, shSTAT3/THP-1 cells were pretreated with this small-molecule and then, stimulated with IFN-γ. In the absence of STAT3, STAT1 became another target for stattic and the percentage of PD-L1-positive cells was decreased considerably (Fig. 4f,g).

Collectively, IFN-γ-induced STAT3 signaling had a minimal role in the upregulation of PD-1 ligands. The negative influence of stattic on PD-L1 expression may be partly explained by its promiscuous effect on STAT1. Nevertheless, further analyses are required to better define the impact of this small-molecule inhibitor of STAT3 on IFN-γ signaling pathway.

## Discussion

Immature facets and heterogeneity of AML and MDS cells are among the major obstacles in developing successful therapy approaches^38^. Even though myeloid maturation is characterized with terminal differentiation and a reduction in proliferative capacity, the well-differentiated subtypes of myeloproliferative diseases have been associated with disease severity and worse prognosis^38,39^. The signaling pathways, which also include STAT3, related to survival, stemness, and immune suppression are essentially involved in the acquisition of therapy resistance and malignant progression^32,38,39^. Correspondingly, ATRA-induced maturation of malignant blasts is currently used as a standard therapy for acute promyelocytic leukemia (APL)^24^. In addition, checkpoint blockade immunotherapies show early promise in myeloid leukemia even though therapeutic modalities are not fully developed^25^.

In AML and MDS, either the level of differentiation, the frequency of CD11b^+^ blasts, or the expression of PD-1 ligands have been described as critical factors that associate with disease progression and limit the benefit from cancer therapy^2,28,38^. Blockade of PD-1 and PD-L1 molecules has been recognized as a promising approach for cancer immunotherapy^9^; however, to date, the efficacy of checkpoint inhibitors in myeloproliferative diseases is not clear. PD-1 ligands, especially PD-L1, are upregulated in response to inflammatory mediators, including IFN-γ^6,7,10^. Therefore, the presence of PD-L1 has been associated with an adaptation process, also known as secondary immune resistance, inaugurated by cancer cells to inhibit anti-tumor immune responses^2,4,8^. As previously demonstrated by our group, myeloid leukemia cells can provide direct costimulation via CD80, CD86, and ICOS-LG molecules, and these malignant blasts are proficient in utilizing elaborate immune evasion strategies including the adaptive resistance and T-cell exhaustion^2,4,5,40^.

Leukemic and dysplastic myeloid cells which respond to IFN-γ, can also upregulate PD-L1 and PD-L2^6,7^. These observations were taken further by this study that identified the CD11b^+^ subpopulation in AML and MDS as the major fraction bearing PD-1 ligands. Increased levels of CD11b and/or advanced maturation-differentiation in myeloproliferative diseases have been regarded as a parameter for bad prognosis^27–30^. From an immunological perspective, the presence of PD-1 ligands may be one of the factors responsible for disease severity. As a renowned anti-tumor cytokine, IFN-γ is highly secreted by type-1 helper T (Th1) cells, cytotoxic T lymphocytes (CTL), and natural killer (NK) cells^8^. Nevertheless, upon activation, these cells upregulate PD-1 receptor and become prone to the negative signals delivered by PD-L1 and PD-L2 which interfere with anti-tumor functions. Therefore, CD11b^+^ malignant blasts possess greater capacity to hijack the PD-1 checkpoint and can more proficiently use the immune evasion mechanism known as the secondary immune resistance. ATRA or D3 treatment did not enhance basal levels of PD-1 ligands but increased responsiveness of these cells to IFN-γ that induced PD-L1 and PD-L2. Possible mechanisms associated with maturation status and IFN-γ responsiveness in AML or MDS remains to be better elucidated; to date, our results show no strong relation between myeloid maturation and  upregulation of IFN-γ receptor (IFNγR1) (data not presented).

The early classification of AML, i.e. French, American, and British (FAB), is based on maturation levels of the cells. Nevertheless, myeloid maturation and differentiation are of the factors that lead to biological heterogeneity in AML and MDS^41^. Accordingly, a marked heterogeneity was observed in the basal expression of PD-1 ligands and in the IFN-γ response of the multiple cell lines and patient samples used. Therefore, our results indicate the differentiation status as a compounding or additive effect on PD-L1 and PD-L2 expression. Considering the success of ATRA treatment that promotes myeloid maturation in leukemia, it might be speculated that our results may propose a combinatory approach with checkpoint blockade and myeloid maturation therapy for AML and MDS. Nevertheless, immune checkpoint inhibitors may result in distinct clinical outcomes in the patients presenting neoplastic cells with different maturation/differentiation status.

IFN-γ signaling activates the transcription factors, especially STAT1 and IRF1, that bind onto the elements in the promoter region of PD-L1 and PD-L2 genes^8,10^. On the other hand, there is compelling evidence that STAT3 pathway can also be triggered by IFN-γ in certain cell types^15,17^. Caldenhoven *et al*. reported the lineage specific activation of STAT3 in neutrophil granulocytes but not in eosinophils, monocytes, and the AML cell line HL-60^16^. Correspondingly, induction of differentiation in HL-60 by ATRA, which results in a neutrophil-like phenotype^31^, significantly enhanced the IFN-γ-induced phosphorylation of STAT3 in the CD11b^+^ subpopulation. Moreover, the AML cell lines and the primary patient samples with a high percentage of CD11b positivity displayed high levels of pSTAT3 when exposed to IFN-γ. The absence of constitutive STAT3 phosphorylation under steady-state conditions indicated a secondary or inducible role for this transcription factor in the oncogenic process in AML or MDS cells studied. Accordingly, STAT3 pathway was strengthened in response to extended exposure to IFN-γ; hence, a dysregulation in the negative feedback mechanisms acting on STAT3 pathway in AML might be perceived^23^. Even though our results showed the direct stimulation of STAT3 in malignant blasts, long-term stimulation with IFN-γ may also result in the secretion of autocrine or paracrine factors reinforcing or modulating this pro-leukemogenic pathway.

STAT1 and STAT3 are identified with opposite activities, anti-tumor vs. pro-tumor; however, these transcription factors display reciprocal regulation, compete for the same promoter binding sequence, and even compensate and functionally substitute each other^42^. Even though the contribution of STAT1 to PD-1 ligand expression is widely acknowledged, the role for STAT3 is disputed potentially due to variations in the experimental systems and the cell types used^11^. The outcomes of IFN-γ exposure appeared to be more complex since both STAT1 and STAT3 pathways were stimulated in malignant myeloid blasts. Direct binding of STAT3 to the promoter region of PD-L1 was demonstrated in melanoma^11^, T-cell lymphoma^43^, dendritic cells^44^, and non-small cell lung cancer cells^44^. Nevertheless, only minor roles for STAT3 were reported on PD-L1 expression in melanoma cell lines when transiently stimulated with IFN-γ^11^. However, to our knowledge, the positive influence of long-term IFN-γ exposure on STAT3 pathway was not reported previously. The induction of myeloid cell maturation/differentiation by ATRA or D3 was one of the factors that supported the expression and activation of STAT3. Although not effective as these agents, IFN-γ can also promote the differentiation of myeloid cells^45^. Therefore, as the duration of IFN-γ exposure was increased, the influence of STAT3 pathway on PD-1 ligand expression might have been pronounced in AML or MDS cells. Further analyses are required to validate possible compensatory and reciprocal interplay between STAT3 and STAT1 on the IFN-γ-induced PD-L1 expression. STAT3 can partly be responsible of basal-level expression of PD-L1, however it had a minor role on the IFN-γ-induced upregulation of this checkpoint ligand. In a study by Garcia-Diaz *et al*., STAT3 was found to be more effective on the promoter activity of PD-L2 than that of PD-L1^11^. Nevertheless, in our study, the impact of STAT3 on PD-L2 was limited potentially because it was constitutively expressed on CD11b^+^ myeloid blasts.

Upregulation of PD-1 ligands and STAT3 transcription factor may serve as distinct strategies that potentiate the pro-tumor actions of IFN-γ^8^. Stattic is recognized as a small-molecule that selectively and directly inhibits both phosphorylation, dimerization, and nuclear translocation of STAT3^36^. Nevertheless, stattic not only inhibited STAT3 pathway but also reduced pSTAT1 levels in a cell-type specific manner. This effect was more prominent in D3-treated HL-60 and shSTAT3/THP-1 which had low levels of STAT3 protein. Therefore, stattic can preferentially interact with STAT3 however STAT1 might have become a second target for the excess molecules. Correspondingly, stattic was previously reported to inhibit IFN-β-induced STAT1 phosphorylation in dendritic cells. Nonetheless, this dual effect of stattic on STAT3 and STAT1 diminished the expression of PD-L1 on the AML and MDS cells. The clinical relevance of this small-molecule inhibitor remains to be elucidated.

**Figure 5:**
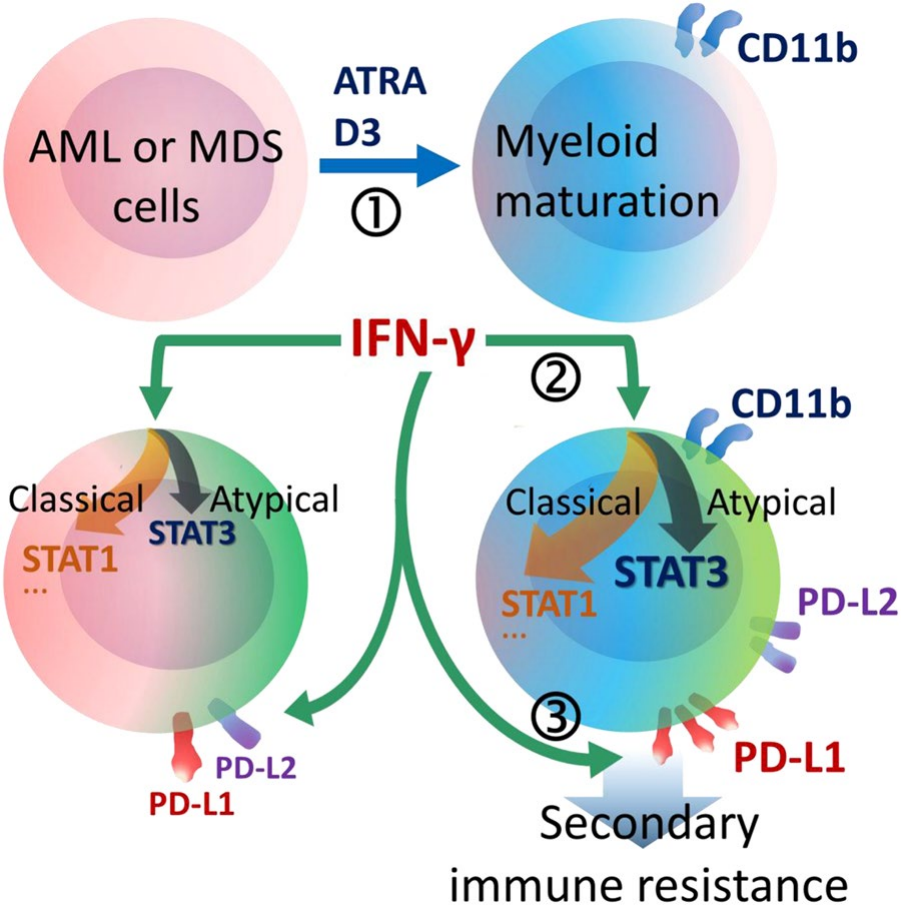
Schematic demonstration of the major findings. (1) Maturation of AML or MDS cells was stimulated by ATRA or D3 treatment and myeloid maturation could be followed by CD11b positivity. (2) ATRA- or D3-treated cells were predisposed to atypical IFN-γ signaling through STAT3 together with classical pathways such as STAT1. (3) In response to IFN-γ, CD11b^+^ mature AML or MDS cells more efficiently upregulated PD-1 ligands, especially PD-L1, which confer to secondary immune resistance.

In conclusion, the CD11b^+^ subpopulation in AML and MDS can remarkably respond IFN-γ and employ adaptive resistance mechanisms comprising STAT3 and PD-1 pathways. Even though the immunotherapy approaches or IFN-γ treatment in myeloproliferative diseases yet to be developed, our findings indicate the unfavorable actions of IFN-γ which may be targeted to support the anti-tumor responses and increase the efficacy of immune intervention therapies in myeloproliferative diseases. Induction of myeloid maturation enhances the responsiveness of AML and MDS cells to IFN-γ. However, these malignant myeloid cells use both STAT3 pathway and PD-1 ligands to survive IFN-γ-mediated immunity and maintain secondary immune resistance (summarized in Fig. 5). Therefore, the differentiation status in myeloid malignancies might be a compounding factor for immune checkpoint blockade therapies.

## Materials and Methods

### Patient and healthy donor samples

Bone marrow (BM) aspirates were obtained from treatment-naïve patients who were diagnosed with AML [n = 19 (9 females, 10 males), median age 53 (min 24–max 77)] or with MDS [n = 16 (5 females, 11 males), median age 62 (min 45–max 79)]. For co-culture experiments, peripheral blood was collected from healthy volunteers. The study was commenced after the approval by Hacettepe University local ethics committee (approval no.: GO 14/120-19), informed consent was obtained from the participants and all methods used were performed in accordance with the relevant guidelines and regulations.

### Cell culture and induction of myeloid maturation

Myeloid leukemia cell lines (KG-1, Kasumi-1, HL-60, THP-1, and U937 with FAB maturation scores 0–1, 1–2, 3, 4–5, and 5, respectively) were either purchased (ATCC, LGC Promochem, Rockville, MD, USA) or obtained by the courtesies of our collaborators and cultured under standard conditions^5^. In order to push forward the myeloid maturation, either myeloid cell lines or the patient-derived BM cells (2 × 10^5^/ml) were incubated with all-trans retinoic acid (ATRA, 1 µM) or 1α,25-Dihydroxyvitamin D3 (D3, 100 nM) (Sigma, St. Louis, MO, USA)^31,32^. The maturation-induced increase in cells’ density was tested with 75%, 50%, 30% and 20% Percoll (Sigma) discontinuous gradient separation (see Supplementary Fig. 1 and Supplementary Fig. 2). Recombinant IFN-γ (50 or 150 ng/mL) and/or IL-6 (10 ng/mL) (R&D, Minneapolis, MN, USA) was used to induce PD-1 ligand expression. In certain experiments, the cells were pre-treated with sub-toxic concentrations of stattic (45 min., 5 µM for THP-1 and ATRA-treated HL-60, 3.75 µM for D3-treated HL-60, 2.5 µM for U937 and 10 or 20 µM for patient BM cells; Santa Cruz Biotechnology, Dallas, Texas, USA). In the experimental setups for autocrine/paracrine factor exclusion, prior to 48h IFN-γ induction, HL-60 and THP-1 cells were treated with monensin (2 µM, 4 h pretreatment; Biolegend, San Diego, CA, USA) in order to inhibit secretory protein transport.

### Flow cytometry and cell sorting

Immunophenotyping was performed with the monoclonal antibodies anti-human-CD4 (SK3), -CD8 (SK1), -CD3 (SK7), -CD13 (L138), -CD14 (M5E2), -CD15 (H198), -CD11b (ICRF44), -CD11c (S-HCL3), -HLA-DR (G46-6), -CD16 (3G8), -CD56 (MY31), -CD66b (G10F5), -CD69 (FN5050), -CD137 (4B4-1) -CD274 (PD-L1; MIH1) (Becton Dickinson, San Jose, CA, USA); -CD273 (PD-L2; 24 F.10C12), -IFNgRa (GIR-94), -CD279 (PD-1; EH12.2H7) (Biolegend). The percentage and median fluorescence intensity (MFI) of positive cells were determined considering the isotype-matched antibody and autofluorescence controls. The myeloid cells with high CD11b expression were positively selected by fluorescence-activated cell sorting (FACS). CD4^+^CD8^−^CD56^−^CD13^−^ helper T (Th) cells and CD4^−^CD8^+^CD56^−^CD13^−^ cytotoxic T lymphocytes (CTLs) were gated and sorted with a purity ≥96%. Immunophenotyping and cell sorting were performed on a BD FACSAria^TM^ II sorter (Becton Dickinson, San Jose, CA, USA).

### Western-Blot analysis

Total cell lysates were run on SDS-PAGE, transferred onto PVDF membranes, and blocked by using previously described standard methods^46^. Primary antibodies (anti-human-total STAT3 (79D7; 1/1500), -phospho-STAT3 (pSTAT3-Tyr705) (D3A7; 1/1500), -total STAT1 (D1K9Y; 1/1500), -phospho-STAT1 (pSTAT1-Tyr701) (58D6; 1/1500), or anti-polyspecies β-actin (D6A8; 1/2500) (Cell Signaling Technology) were used together with appropriate HRP-conjugated secondary antibodies. Density of each protein band was normalized to the corresponding ECL chemiluminescence from β-actin values (Gel Logic 1500, Rochester, NY, USA).

### Lentiviral transduction

Lentiviral vector plasmids, pSIH1-puro-control shRNA, pSIH1-puro-STAT3 shRNA, EF.STAT3C.Ubc.GFP, EF.STAT3DN.Ubc.GFP, and the plasmids used for lentivirus packaging were obtained from Addgene^33,37,47,48^. The procedures used for lentivirus production and transduction of THP-1 cells (3 × 10^5^ cells/mL; MOI = 2) were modified from previously described methods^49^. The genetically-modified cells were enriched by puromycin (1 μg/mL) selection or GFP^+^ cells were purified by FACS.

### Reverse transcription-polymerase chain reaction (RT-PCR)

RNA was isolated (QIAamp RNA Mini Kit, QIAGEN, Maryland, MD, USA), treated with RNase-free DNase (RNA Clean & Concentrator^TM^, Zymo Research, Irvin, CA, USA) and converted to cDNA (RevertAid^TM^ First Strand cDNA Synthesis Kit, Fermentas, Vilnius, Lithuania). Primer oligonucleotides for STAT3, forward 5′-TCACGCCTTCTACAGACTGCAG-3′, reverse 5′-TCCGGACATCCTGAAGGTGCTG-3′; PD-L1, forward 5′-GACATGTCAGGCTGAGGGCT-3′, reverse 5′-TGATTCTCAGT GTGCTGGTCACA-3′; PD-L2, forward 5′-TGAGGTAGAGCTCACCTGCCA-3′, reverse 5′-ACACAGCTGAAGTTTCTGCCAG-3′; β-actin, forward 5′-CTGGAACGGTGAAGG TGACA-3′, reverse 5′-AAGGGACTTCCTGTAACAATGCA-3′ were used in conventional or semi-quantitative real-time (SsoAdvanced Universal SYBR Green Supermix, Bio-Rad, Hercules, CA, USA) PCRs. In real-time PCR, the relative difference in mRNA levels was determined with comparative Ct (2^−ΔΔCt^) formula considering β-actin and control (untreated) sample expression data for normalization. Conventional PCR products were documented under UV light after agarose gel electrophoresis and ethidium bromide staining.

### Functional assays on T-cells

Purified Th cells or CTLs (25 × 10^3^/200 µL) were co-cultured with myeloid leukemia cells at 1:0.125 or 1:0.25 ratio. Leukemia cells were treated with IFN-γ for 48 h and then co-cultured for 72 h with anti-CD3-stimulated (HIT3a; 25 ng/mL) T-cells labelled with carboxyfluorescein succinimidyl ester (CFSE, 5 µM; CellTrace^TM^, Invitrogen, Eugene, OR, USA). PD-1-Fc recombinant protein (4 µg/mL) (R&D) or IgG isotype control (MOPC-21) was added into the co-cultures. The dilution in the fluorescence intensity of CFSE (initial MFI/resulting MFI) was analyzed.

### Statistical analysis

Data presented are typical of at least three independent experiments. Arithmetic mean ± standard deviation (SD) or standard error (SE) are used for the graphics shown. Statistical difference between the groups was evaluated with the analysis of variance (ANOVA) with post-hoc tests or Student’s paired or unpaired t test. Differences with a *P value* ≥ 0.05 were regarded as statistically significant.

## Supplementary information

Supplementary data

## Data Availability

For original data, please contact gunese@hacettepe.edu.tr.
