# Supplementary material for: Myeloid maturation potentiates STAT3-mediated atypical IFN-γ signaling and upregulation of PD-1 ligands in AML and MDS
Source: Sci Rep. 2019 Aug 12;9:11697. doi: 10.1038/s41598-019-48256-4 (PMC6691003; doi:10.1038/s41598-019-48256-4)
Supplement: Supplementary file 1 — Supplementary data [file 41598_2019_48256_MOESM1_ESM.pdf]

Myeloid maturation potentiates STAT3-mediated atypical IFN-γ signaling and upregulation of PD-1 ligands in AML and MDS

Digdem Yoyen-Ermis, Gurcan Tunali, Ece Tavukcuoglu, Utku Horzum[1], Didem Ozkazanc, Tolga Sutlu, Yahya Buyukasik, and Gunes Esendagli

Supplementary Information

Supplementary Figures

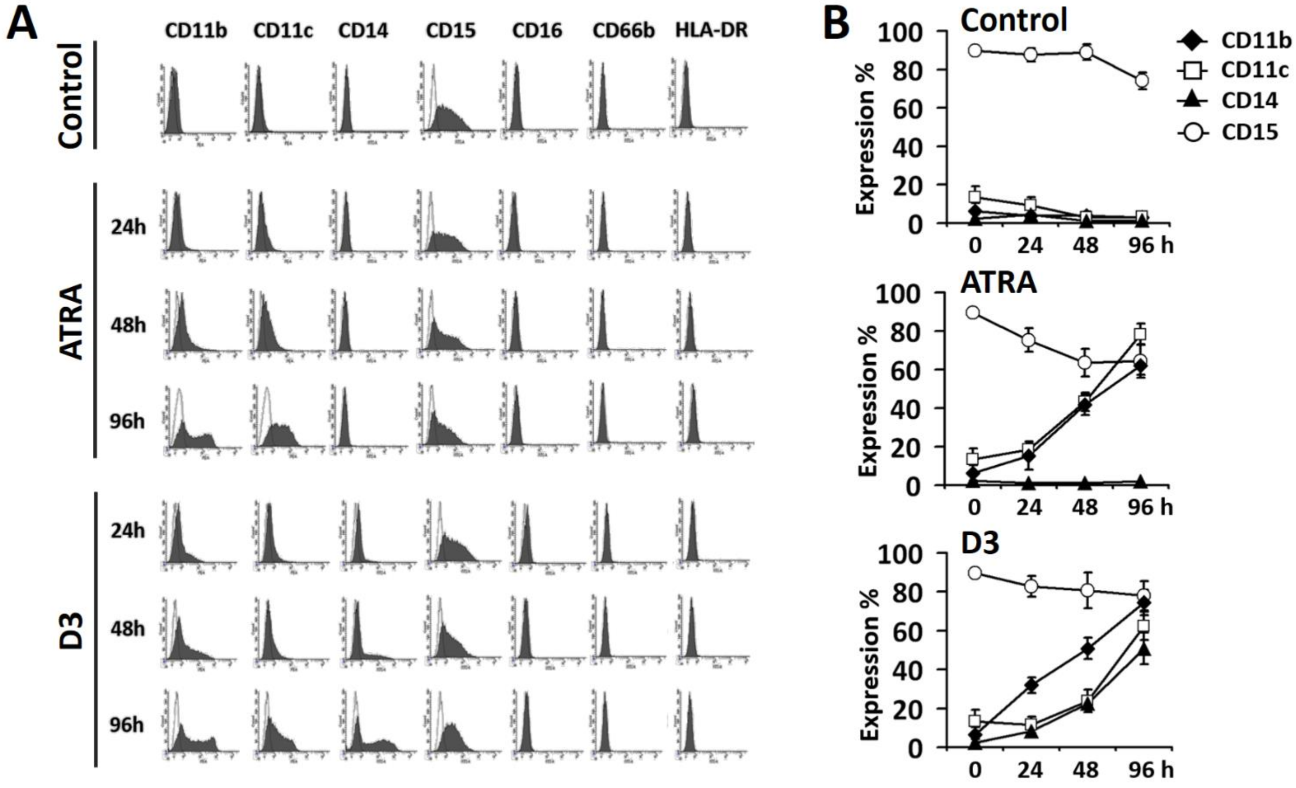

Supplementary Figure 1. Expression of myeloid maturation markers upon ATRA or D3 treatment. Results obtained with HL-60 cell line are shown. **A)** Representative flow cytometry histograms of the surface molecules associated with myeloid maturation (empty histogram, staining with isotype-matched control antibody; filled histogram, staining with specific antibody). **B)** The change in the percentage of cells positive for CD11b, CD11c, CD14, or CD15 in response to ATRA of D3 through 96h-long incubation period.

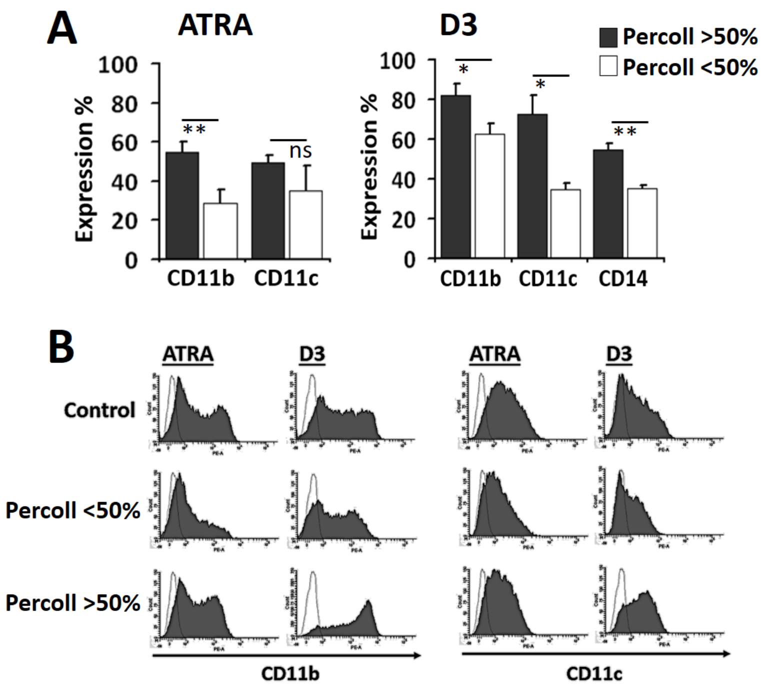

Supplementary Figure 2. Confirmation of myeloid maturation with cell density. Results obtained with HL-60 cell line are shown. **A)** Distribution of ATRA- or D3-treated cells carrying CD11b, CD11c, or CD14 over or below 50% Percoll fraction. **B)** Representative flow cytometry histograms for the cells expressing CD11b or CD11c are demonstrated (empty histogram, staining with isotype-matched control antibody; filled histogram, staining with specific antibody). (**P*<0.05, ***P*<0.01; *n=6*; ns, not significant).

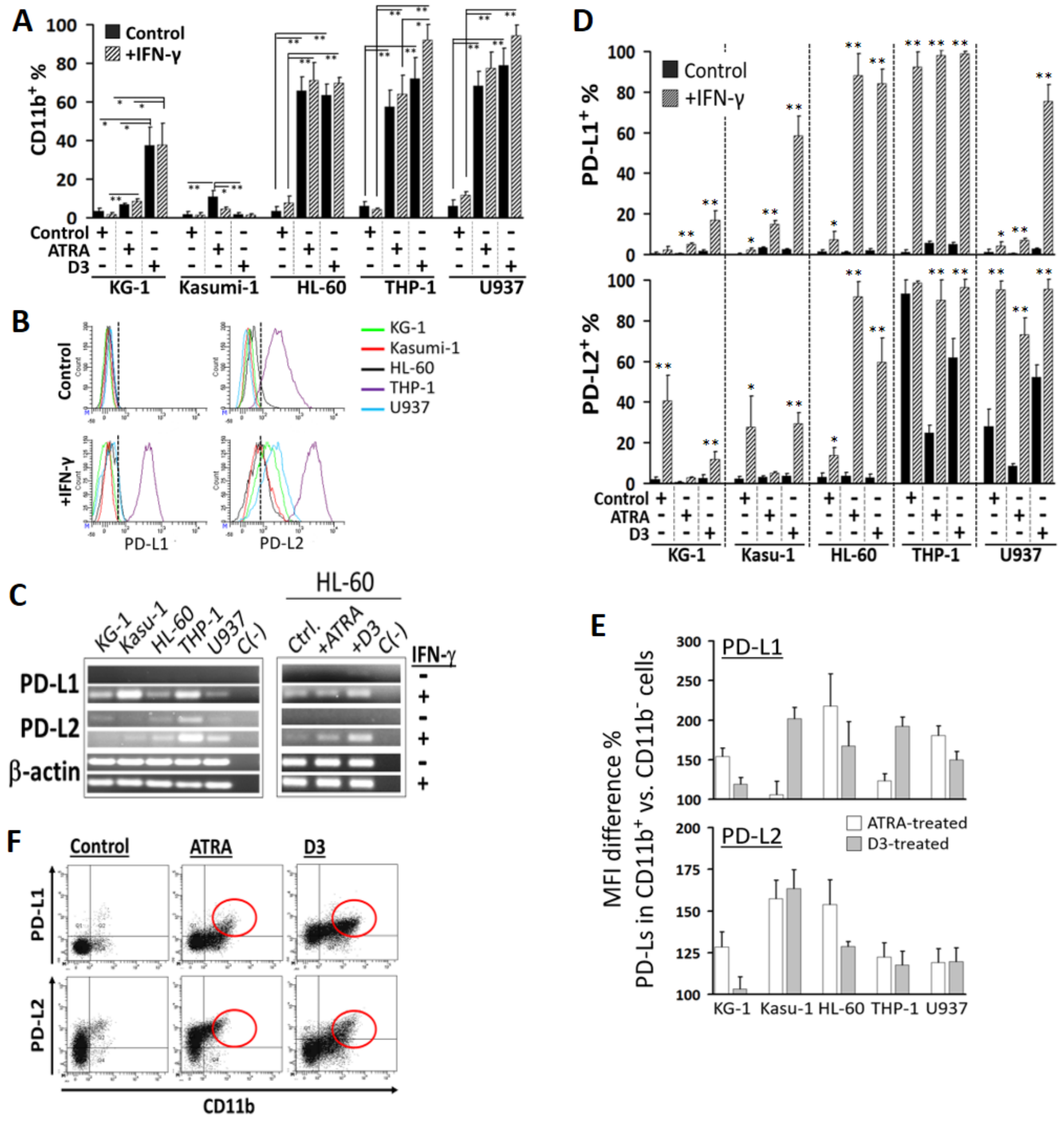

Supplementary Figure 3. CD11b and PD-1 ligands' in myeloid leukemia cells under the influence of ATRA- or D3-induced maturation. A) The cell lines, which are aligned in the x-axis according to their maturation characteristics according to FAB classification, were incubated with ATRA or D3 for 96h and/or with IFN-γ for 48h. The percentage of CD11b$^+$ cells was determined by flow cytometry. **B)** Representative overlay histograms for basal level and IFN-γ-induced expression of PD-L1 and PD-L2 on myeloid leukemia cells with different FAB classification/maturation properties. **C)** Induction of PD-1 ligand gene expression in myeloid leukemia cell lines in response to IFN-γ (32h). The right-panel shows the results from HL-60 cells that were treated with ATRA or D3. B-actin is used as house-keeping gene. Representative agarose gel electrophoresis results from conventional PCR are shown. (Ctrl., control; C(-), dH_2O negative control). **D)** Myeloid cell lines were pretreated with ATRA or D3 as an internal control for myeloid maturation that increase the frequency of CD11b positive cells. Following the incubation with IFN-γ, the percentage of PD-L1- or PD-L2-positive cells were plotted. **E)** Difference in the levels of IFN-γ-induced PD-1 ligands on CD11b$^+$ subpopulation was calculated in comparison to CD11b$^-$ cells which were pretreated with ATRA or D3. **F)** Co-expression of CD11b and PD-L1 or PD-L2 on the ATRA- or D3- treated HL-60 cells that were stimulated with IFN-γ for 48h. Red

ellipses indicate the PD-L1 or PD-L2 positivity of the population with high CD11b expression. (*P<0.05, **P<0.01; for cell lines, $n \geq 3$; patient samples, $n=30$; MFI, median fluorescence intensity).

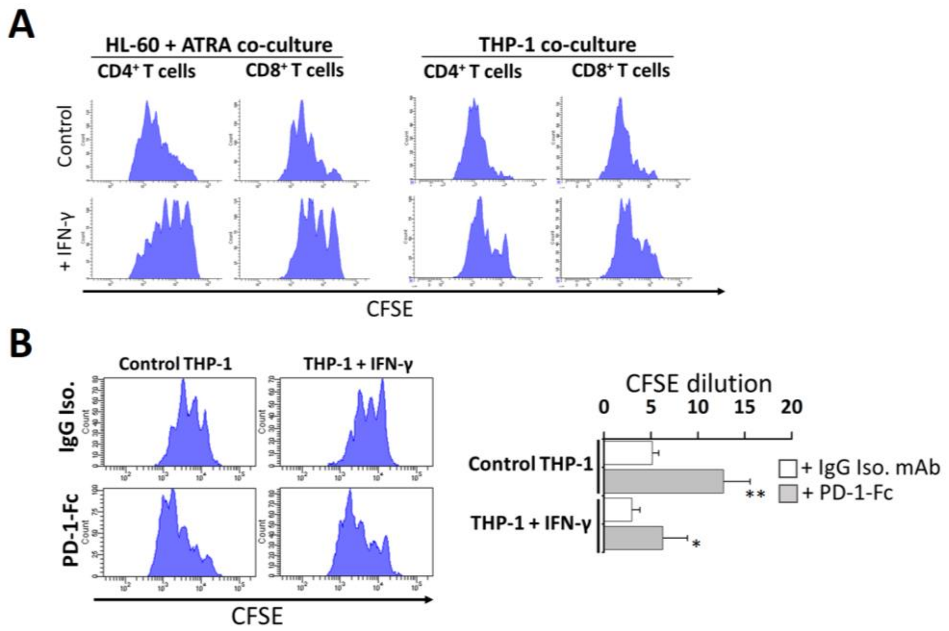

Supplementary Figure 4. T cell proliferation in the presence of IFN-γ-induced myeloid leukemia cells. Freshly isolated CD4+ or CD8+ T cells were labelled with CFSE, stimulated with anti-CD3 mAb and co-cultured with ATRA-treated HL-60 or THP-1 cell lines (myeloid cell:T cell co-culture ratio 0.125:1 or 0.5:1) that were previously induced with IFN-γ. **A)** Representative flow cytometry histograms for CFSE fluorescence out of three independent experiments are given. **B)** The CFSE-based proliferation assays were performed in the presence of isotype-matched control IgG (IgG Iso.) or PD-1-Fc protein that blocks PD-1 ligands. Left panel shows representative results from T cells co-cultured with THP-1 or IFN-γ-induced THP-1 cells. Quantification of T cell proliferation as CFSE dilution is given in the right panel. (*P<0.05, **P<0.01; $n=3$).

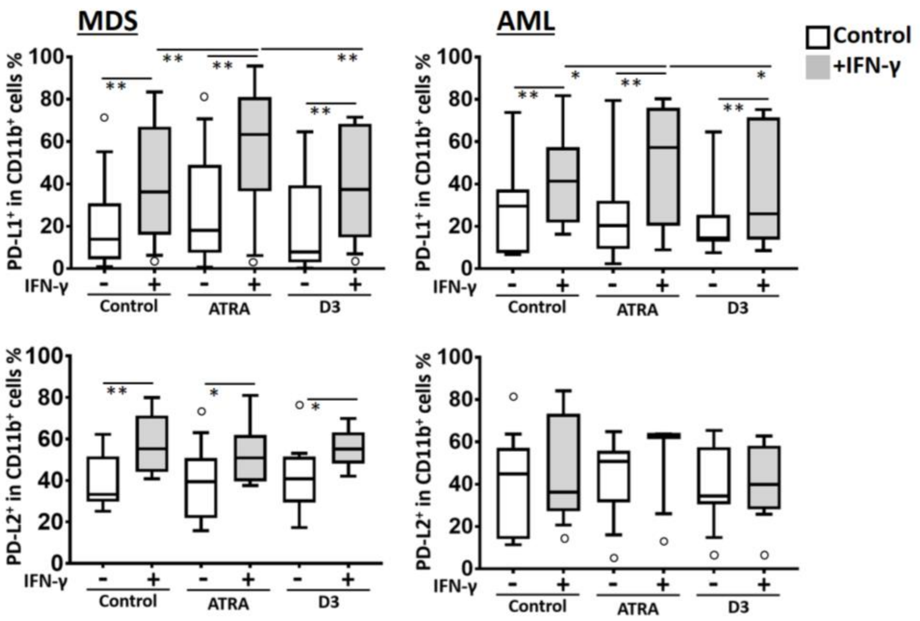

Supplementary Figure 5. The effect of ATRA or D3 treatment on PD-1 ligand expression in patient samples. The myeloid blasts from MDS (left panels; *n=16*) and AML (right panels; *n=10*) patients' bone marrow aspirates were pretreated with ATRA or D3 (96h); then, the percentage of IFN-γ-induced PD-L1 (upper panels) or PD-L2 (lower panels) expression (48h) on the $CD11b^+$ population was determined. Outliers are shown as empty circles. (**P*<0.05, ***P*<0.01).

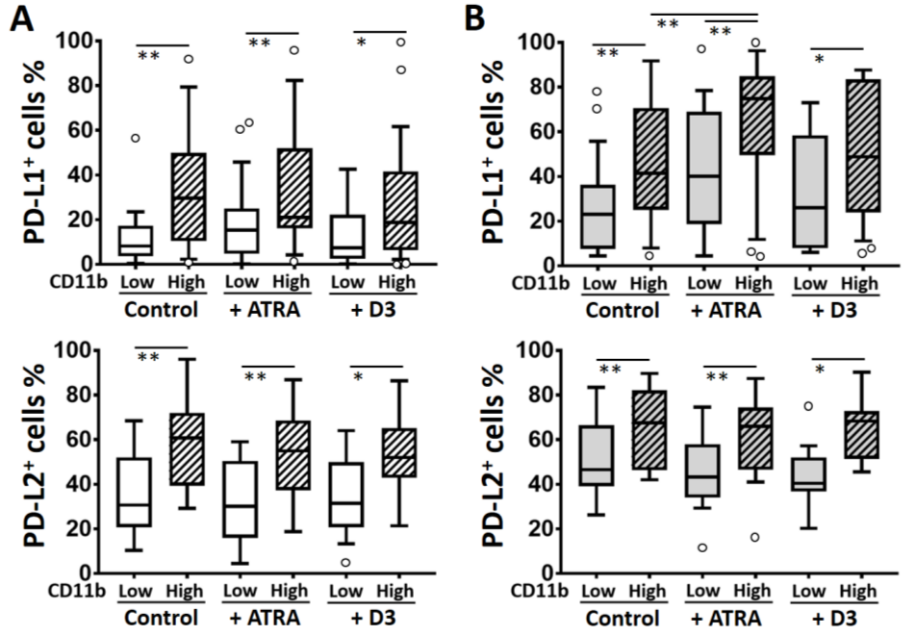

Supplementary Figure 6. High-level CD11b on the MDS and AML patient-derived myeloid blasts is an indicator of increased capacity for PD-1 ligand expression. Bone marrow aspirates from the patients were stimulated with ATRA or D3 and/or IFN-γ. Then, $CD11b^+$ MDS and AML cells were gated into two subpopulations according to low and high CD11b fluorescence intensity and the percentage of cells carrying PD-L1 and PD-L2 was analyzed by flow cytometry. Box plots show the results from **(A)** unstimulated and **(B)** IFN-γ-stimulated patients samples. (**P*<0.05, ***P*<0.01; patient samples, *n=30*).

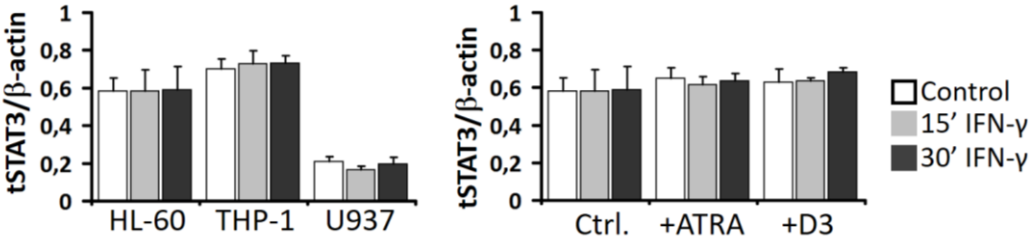

Supplementary Figure 7. Total STAT3 protein levels were not significantly changed upon stimulation with IFN-γ for 15 or 30 minutes. Bar graphs display the semi-quantitative data from Western-Blot analysis calculated by normalizing the band intensities of total STAT3 to β-actin. Right panel show the results from HL-60 cells that were matured with ATRA or D3 for 96h. (*n=3*).

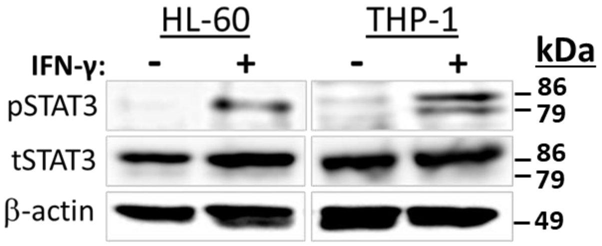

Supplementary Figure 8. Induction of pSTAT3 by IFN-γ in myeloid leukemia cells in the presence of monensin, a secretory protein transport inhibitor, in order to exclude the influence of paracrine or autocrine factors influence on STAT3 induction. HL-60 and THP-1 cells were pretreated with monensin for 4h and followed by 48h incubation with IFN-γ in the presence of monensin. Total STAT3 and Tyr705 phosphorylated pSTAT3 levels of were evaluated by Western-Blot.

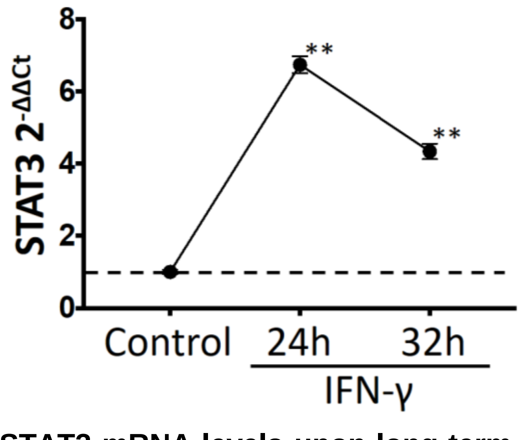

Supplementary Figure 9. STAT3 mRNA levels upon long-term IFN-γ induction of $CD11b^+$ cells from healthy donors' peripheral blood. The change in STAT3 mRNA levels at 24 and and 32h was determined by real-time RT-PCR. The dashed line crossing at $2^{-\Delta\Delta Ct}=1$ indicates equal expression level with control (0h) cells. (***P*<0.01, n=3).

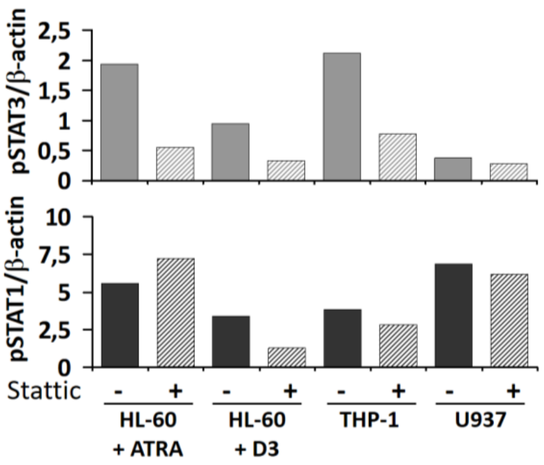

Supplementary Figure 10. The change in pSTAT3 (Tyr705) and pSTAT1 (Tyr701) levels in IFN-γ-stimulated (15 min.) myeloid cells that were pretreated with stattic. Bar graphs display the semi-quantitative data from Western-Blot analysis calculated by normalizing the band intensities of pSTAT3 or pSTAT1 to β-actin. In order to induce myeloid maturation HL-60 cells were treated with ATRA or D3 for 96h. (*n=3*).

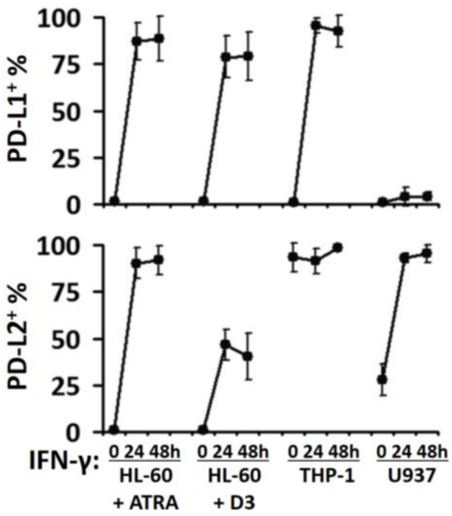

Supplementary Figure 11. The change in myeloid cells positivity for PD-L1 and PD-L2 in response to IFN-γ treatment for 24h or 48h were studied by flow cytometry. In order to induce myeloid maturation HL-60 cells were treated with ATRA or D3 for 96h. (*n=3*).

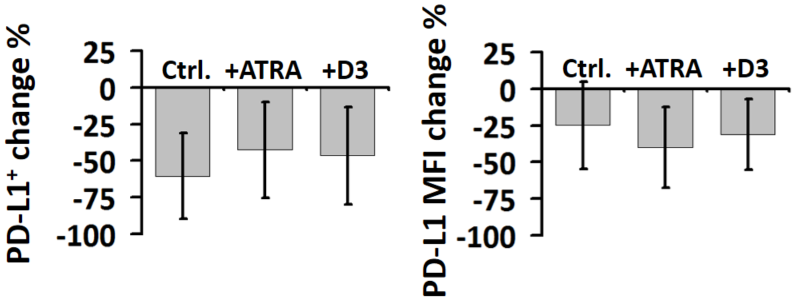

Supplementary Figure 12. Pretreatment with stattic downregulates IFN-γ-induced PD-L1 expression in patient derived CD11b⁺ myeloid blasts. Bone marrow aspirates from the control, ATRA- or D3-treated AML or MDS patients were pretreated with stattic (45 min) and then, stimulated with IFN-γ for 24h. The expression of PD-L1 was evaluated on myeloid blasts gated on CD11b. The change in the percentage of PD-L1⁺ cells (left panel) and PD-L1 surface levels (right panel) were calculated. For each individual, the data from the stattic-treated cells were normalized to those from controls that were not treated with stattic. (*n=7*; MFI, median fluorescence intensity).

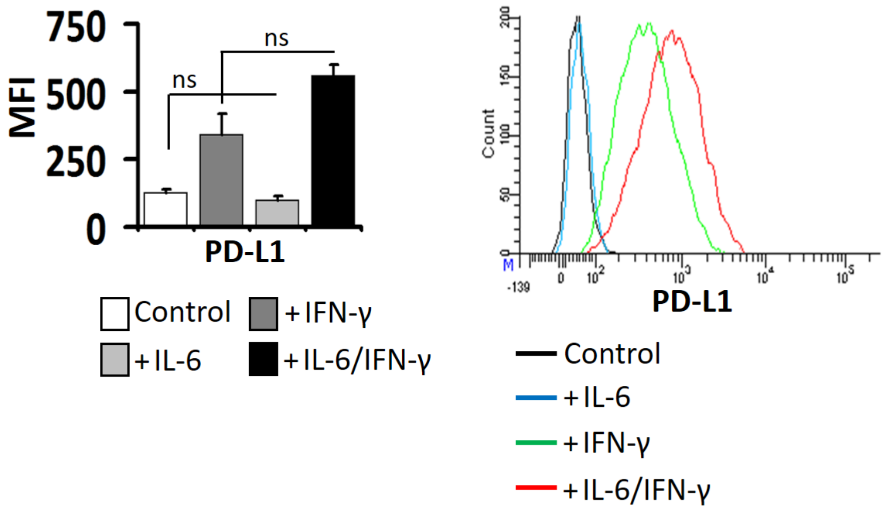

Supplementary Figure 13. IL-6 as a common cytokine inducing STAT3 pathway did not increase PD-L1 expression. THP-1 cells were incubated with recombinant IL-6, IFN-γ or combination of both cytokines for 24h and the levels of PD-L1 were assayed by flow cytometry. Median fluorescence intensity (MFI) bar graphs and flow cytometry overlay histograms are demonstrated. (n=3; ns, not significant).
